# Characterization of Liposarcoma Cell Lines for Preclinical and Biological Studies

**DOI:** 10.1155/2012/148614

**Published:** 2012-07-14

**Authors:** Eva W. Stratford, Russell Castro, Jeanette Daffinrud, Magne Skårn, Silje Lauvrak, Else Munthe, Ola Myklebost

**Affiliations:** ^1^Cancer Stem Cell Innovation Centre and Department of Tumor Biology, Institute of Cancer Research, Oslo University Hospital, The Norwegian Radium Hospital, P.O. Box 4953, Nydalen, 0424 Oslo, Norway; ^2^Department of Molecular Bioscience, University of Oslo, P.O. Box 1041, Blindern, 0316 Oslo, Norway

## Abstract

Liposarcoma cell lines represent *in vitro* models for studying disease mechanisms at the cellular level and for preclinical evaluation of novel drugs. To date there are a limited number of well-characterized models available. In this study, nine immortal liposarcoma cell lines were evaluated for tumor-forming ability, stem cell- and differentiation potential, and metastatic potential, with the aim to generate a well-characterized liposarcoma cell line panel. Detailed stem cell and differentiation marker analyses were also performed. Five of the liposarcoma cell lines were tumorigenic, forming tumors in mice. Interestingly, tumor-forming ability correlated with high proliferative capacity *in vitro*. All the cell lines underwent adipocytic differentiation, but the degree varied. Surprisingly, the expression of stem cell and differentiation markers did not correlate well with function. Overall, the panel contains cell lines suited for *in vivo* analyses (LPS141, SA-4, T778, SW872, and LISA-2), for testing novel drugs targeting cancer stem cells (LPS141) and for studying tumor progression and metastasis (T449 and T778).

## 1. Introduction

Liposarcoma is categorized into three main subtypes; well-differentiated/dedifferentiated liposarcomas (WD/DDLPSs), myxoid/round-cell liposarcomas, and undifferentiated high-grade pleomorphic liposarcomas (reviewed [1]). WDLPSs are local low-grade tumors, which do not metastasize unless they dedifferentiate. Progression to dedifferentiated liposarcoma (DDLPS) occurs in ~25% of WDLPS [2], but the process is poorly understood. Ten to twenty % of DDLPS undergo metastasis and overall mortality is 50–70% [2–4]. Both WDLPS and DDLPS have unique molecular characteristics, containing supernumerary ring and/or giant rod chromosomes containing amplified segments from 12q13–15 [5, 6]. The most common treatment for LPS is surgery, sometimes combined with radiotherapy and chemotherapy. Sensitivity to chemotherapy varies greatly between subtypes, with WD/DDLPS responding poorly (reviewed [1]). Well-characterized model systems are required for improved understanding of the molecular processes driving liposarcoma genesis, such as tumor formation, dedifferentiation, and metastasis and also for preclinical testing of novel therapies, but there is a lack of models, with only 1 LPS cell line (SW872) available commercially. However, a number of immortal LPS cell lines have been generated [7–11] and a small number of LPS cell lines and xenografts have been included in recent characterizations [12, 13]. This study initiates an effort in establishing an extended, well-characterized collection of LPS models, with emphasis on WD/DDLPS.

## 2. Materials and Methods

### 2.1. Cell Lines and Culturing

SW872 (undifferentiated LPS) and SA-4 (classified as “liposarcoma”) were both purchased from ATCC. LISA-2, generated from a metastasis of a poorly differentiated liposarcoma [10], was provided by Dr. Möller. FU-DDLS-1 and LPS141, both established from DDLPS tumors [7, 9], were gifts from Dr. Nishio and Dr. Fletcher, respectively. GOT-3, generated from a recurrence of a myxoid variant of a WDLPS [8], was provided by Dr. Åman. T449 and T778 were established from a primary WDLPS and its recurrence, respectively, and T1000 (from a WDLPS recurrence) were all gifts from Dr. Pedeutour. The cells were maintained in RPMI-1640 (Lonza); 10% fetal bovine serum (FBS) (PAA laboratories Gmbh, Pashing, Austria); GlutaMAX and penicillin/streptomycin (both from Life Technologies, Carlsbad, CA). Short-tandem-repeat- (STR-) DNA profiling of 15 loci and amelogenin was performed (Genetica DNA Laboratories, OH, USA). For SW872, the obtained STR-DNA profile was compared with the ATCC database, while T449 and T778 were compared to each other. Amelogenin status was compared to the patient gender, when known. Primary human mesenchymal stroma cells (hMSCs) (obtained from the hip of a healthy female donor), provided by Dr. Kvalheim and Mr. Wang (Norwegian Radium Hospital), were cultured in minimum essential medium alpha medium (Life Technologies); 20% FBS; GlutaMAX and penicillin/streptomycin. Informed consent and sample collection were approved by the Ethical Committee of Southern Norway (S-90128).

### 2.2. Proliferation Assay

Cellular proliferation rates were determined by live cell imaging (IncuCyte, Essens Bioscience, Birmingham, UK). More specifically, an equal number of cells were plated in 96-well format and phase contrast photographs were taken automatically every second hour for the duration of the experiment. The data was presented as cell confluence over time.

### 2.3. Adipocytic Differentiation Assays

Adipocytic differentiation and oil red O staining was performed as described in [14]. hMSCs were cultured for 21 days and LPS cell lines for 10–15 days.

### 2.4. Colony Assay

One thousand single cells were plated in methocult (catalogue number 04100, Stem Cell Technology, Grenoble, France) supplemented with stem cell medium (final concentration 1x B27; penicillin/streptomycin; glutaMAX (all Life Technologies); 20 ng/mL basic fibroblast growth factor; 20 ng/mL epidermal growth factor (both PeproTech, Stockholm, Sweden); as recommended by Stem Cell Technology. Uniform colonies (>50 *μ*m) were counted using GelCount (Oxford Optronix, Oxford, England).

### 2.5. RNA Expression Analyses

qRT-PCR was performed as described previously [14] using one of the primers: *CEBPB*, *PPARG*, *CEBPA*, *FABP4*, NANOG, OCT4,   SOX2, *TBP,* or GAPDH.

### 2.6. Protein Expression Analyses of Stem Cell Markers

Aldefluor assay and analyses of cell surface antigen expression of CD44, CD90, CD73, CD105, and CD133/2(293C) were performed by flow cytometry, as described previously.

### 2.7. *In  Vivo* Tumorigenicity

Animal experiments were performed according to protocols approved by the National Animal Research Authority in compliance with the European Convention of the Protection of Vertebrates Used for Scientific Purposes (approval ID1499 or 3275, http://www.fdu.no/). 1 × 10^6^ cells were injected subcutaneously into both flanks of locally bred NOD/SCID IL2R-gamma-0 (NOD/SCID) mice. Cell viability was confirmed prior to injection.

### 2.8. Migration and Invasion Assay

Twenty-five thousand cells in RPMI-1640 containing 1% FBS were added to chambers containing membranes with 8-micron pores (BD Biosciences) through which the cells can migrate. For invasion assays, similar chambers covered with matrigel were used (BD Biosciences). Chemoattractant was RPMI-1640 containing 10% FBS. Cells were incubated in a humidified incubator at 37°C for 22 hours. Nonmigratory/noninvasive cells were removed by “swabbing” and migratory/invasive cells were fixed and stained with Hemacolor (Merck KGaA, Darmstadt, Germany) and counted.

## 3. Results

### 3.1. Proliferative Capacity

**Figure 1:**
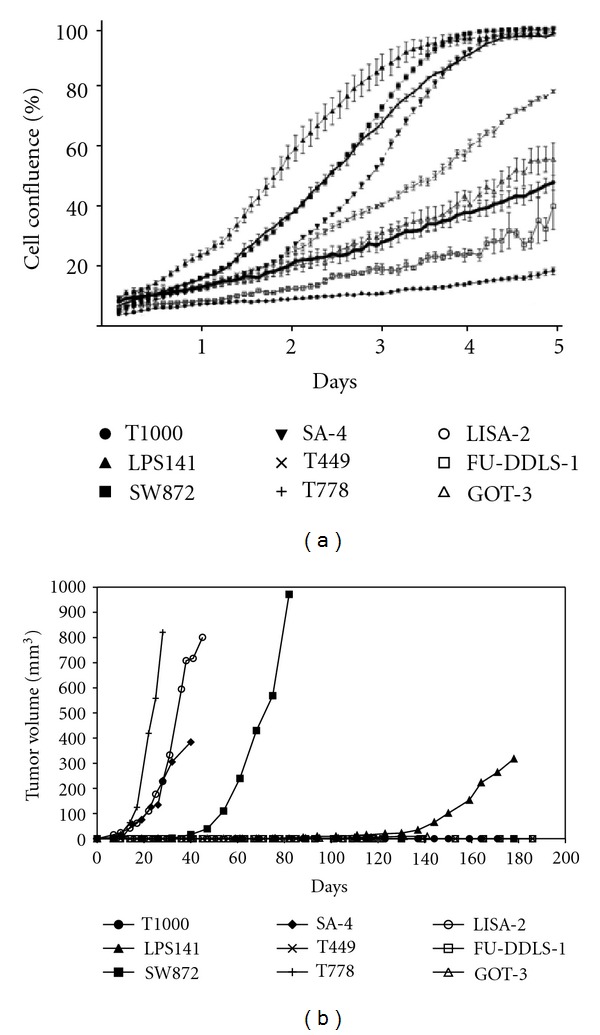
Proliferation capacity and tumor-forming ability of the LPS cell lines. (a) The proliferative capacity was determined by live cell imaging (cell confluence versus time). (b) Tumor formation was determined by injecting 1 × 10^6^ cells into NOD-SCID mice and measuring growth over a 6-month period.

We evaluated the proliferative capacity of the LPS cell lines by live cell imaging. LPS141, SA-4, T778 and SW872 displayed relatively high proliferative capacity and LISA-2, T449, GOT-3, FU-DDLS-1, and T1000 displayed lower proliferative capacity (Figure 1(a)).

### 3.2. Tumorigenicity *In  Vivo*

To determine the tumor-forming ability of the cells, we injected 1 × 10^6^ cells from each line subcutaneously into both flanks of 3 NOD SCID mice. Experiments were repeated if no tumors were obtained. LPS141, SA-4, T778, SW872, and LISA-2 all formed tumors by 6 months (Figure 1(b)). SA-4, T778, and LISA-2 formed tumors rapidly and LPS141 formed tumors very slowly. The other cell lines were not tumorigenic (Table 1). More specifically, GOT-3 and T449 formed small, flat growths which failed to increase significantly in size.

### 3.3. Basal Differentiation Status

**Figure 2:**
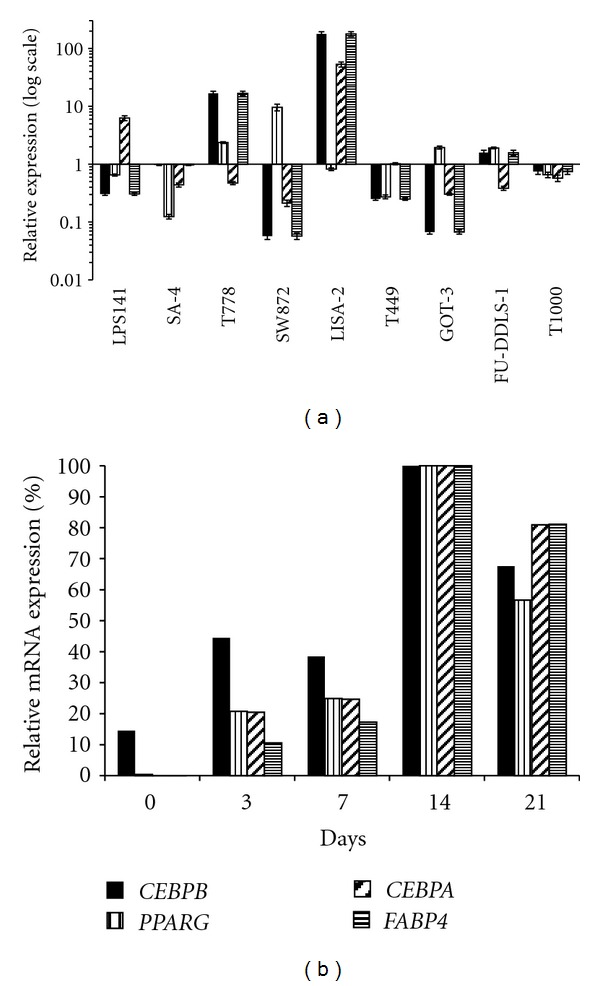
Expression of adipocytic differentiation markers. (a) The basal mRNA levels of *CEBPB*, *CEBPA*, *PPARG, *and *FABP4* normalized to *TBP* were determined in the 9 LPS cell lines and presented relative to the average expression of each gene (log scale). (b) Primary hMSCs were subject to adipocytic differentiation for 21 days. *CEBPB*, *CEBPA*, *PPARG, *and *FABP4* (normalized to *GAPDH*) were determined by qRT-PCR at indicated time points. Data is presented relative to day 14 (set as 100%).

**Figure 3:**
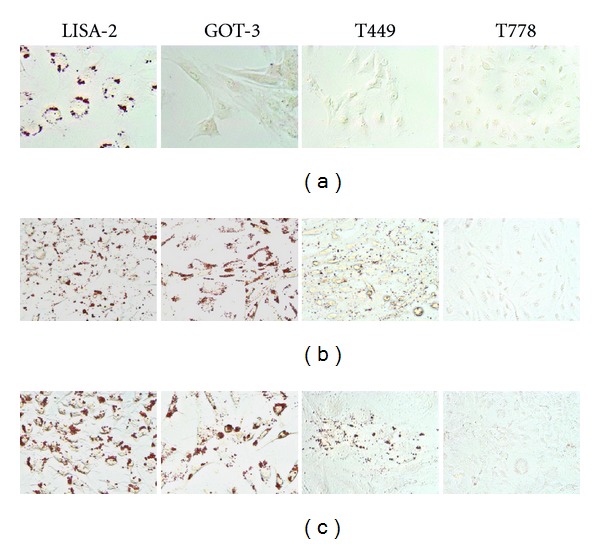
Adipocytic differentiation potential of the LPS cell lines. Shown is oil red O staining of lipid-containing vacuoles in cells during (a) logarithmic growth in normal cell culture medium (basal differentiation status), (b) high cell density growth for 10 days in normal cell culture medium (spontaneous differentiation potential), (c) high cell density growth for 10 days in adipocytic differentiation medium (induced differentiation potential).

Early adipocytic differentiation is partly regulated by the transcription factors C/EBP*β*, C/EBP*α*, and PPAR*γ*, which are used as differentiation-markers [15–18]. C/EBP*α* and PPAR*γ* activate transcription of adipocyte-specific genes, such as *FABP4*, thus used as a marker of later-stage adipogenesis [19]. Basal mRNA levels of *CEBPB*, *CEBPA*, *PPARG,* and *FABP* were determined in the LPS cell lines by qRT-PCR, normalized to TBP, and presented as relative to the average expression of the individual genes in the LPS cell lines, indicating the degree of adipocytic differentiation (Figure 2(a)). We subsequently determined the mRNA expression of the same genes in primary hMSCs before adipocytic differentiation (day 0), during (day 3/7) and after full differentiation (day 14/21). The data was normalized to GAPDH and presented as relative to day 14 (set as 100%) (Figure 2(b)). LISA-2 expressed high mRNA levels of all 4 markers, mimicking the expression pattern of differentiated hMSC. T778 expressed only *CEBPB* and *FABP4* strongly, a pattern which could not be correlated with hMSC. The remaining cell lines expressed transcript levels of all 4 markers relatively similar to the undifferentiated hMSCs (approximately average expression levels). TBP was chosen as a calibrator for the cell lines since TBP was expressed at similar levels in all the cells lines, while GAPDH expression varied greatly between the different LPS cell lines. GAPDH was chosen as a calibrator for the hMSC differentiation experiment since GAPDH was expressed at equivalent levels throughout differentiation while TBP was expressed at very low levels in primary hMSC. Only LISA-2 appeared well differentiated by oil red O staining during logarithmic growth under standard cell culture conditions (Figure 3(a)).

### 3.4. Adipocytic Differentiation Potential

All the cell lines, except T778, underwent spontaneous adipocytic differentiation when cultured in normal medium at high cell density for 10 days, as indicated by positive oil red O staining (Figure 3(b)). The presence of adipocytic differentiation medium induced more and larger lipid-containing vacuoles in a higher percentage of the cells (Figure 3(c)). T778 did not differentiate, but did contain fat droplets following extended induction (15 days) (data not shown).

### 3.5. Stem Cell Markers

**Figure 4:**
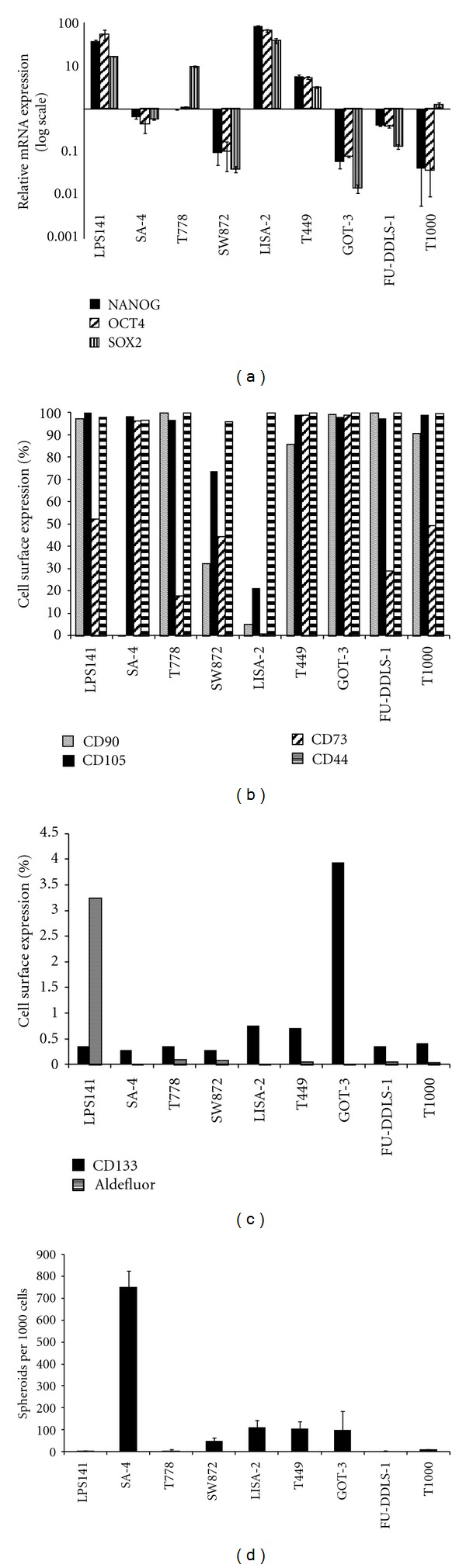
Stem cell phenotypes of the LPS cell lines. (a) NANOG, OCT4, and SOX2 (normalized to *TBP*) were determined by qRT-PCR and presented relative to the average expression of each gene (log scale). (b) Cell surface protein expression of CD90, CD105, CD73, and CD44 was determined by flow cytometry. (c) Expression of CD133 and Aldefluor activity was determined by flow cytometry. (d) Colony-forming ability is presented as colonies >50 *μ*m generated from 1000 cells, following 14 days culturing in semi-solid medium.

We determined the expression of the pluripotency factors OCT4, SOX2, and NANOG (Figure 4(a)) and found all three genes expressed at high levels in LPS141 and LISA-2 (more than 10-fold above average), while expression was intermediate in T449. T778 expressed high levels of SOX2 only. The other cell lines displayed low mRNA expression of all three genes.

We also determined the cell surface expression of CD90, CD105, CD73, CD44 (Figure 4(b)), and CD133 (Figure 4(c)) and measured aldehyde dehydrogenase (ALDH) activity (Figure 4(c)). CD90 was expressed in almost every cell in most lines, except SW872 and LISA-2, which expressed CD90 in a smaller population of cells (5–30%) and SA-4 which did not express CD90 (<0.1%). CD105 and CD44 were ubiquitously expressed in all the cell lines, except LISA-2 and SW872, which expressed CD105 only in a population of the cells. CD73 expression varied between the cell lines. All the lines expressed CD133 in a subpopulation of cells. More specifically, 4% of GOT-3 and less than 1% of the other lines expressed CD133. LPS141 displayed significant Aldefluor activity (>3% of the cells), while the other lines contained subpopulations of <0.2%  Aldefluor^high^.

SA-4 formed colonies efficiently (75%) (Figure 4(d)), while LISA-2, T449, and GOT-3 displayed intermediate capacity (>10%). LPS141, T778, SW872, FU-DDLS-1, and T1000 had very low colony-forming ability (<10%).

### 3.6. Metastasis-Associated Phenotypes

**Figure 5:**
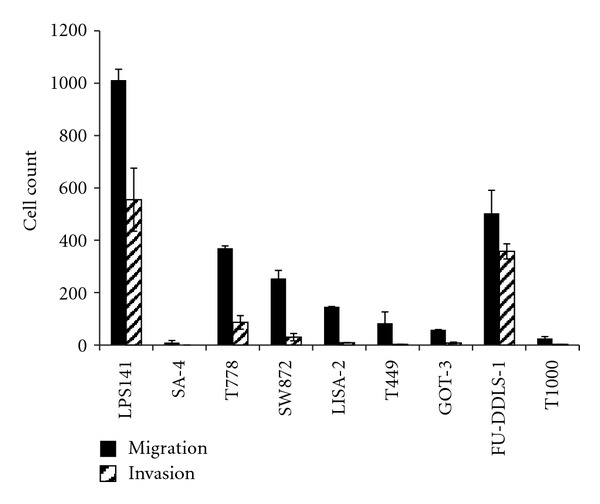
*In  vitro* migration and invasion potential of the LPS cell lines. Cells were incubated for 22 hours.

LPS141, T778, and FU-DDLS-1 displayed high capacity to migrate during 22-hour incubation in Boyden Chambers. SW872, LISA-2, T449, and GOT-3 were also migratory, although to a lesser degree. SA-4 and T1000 displayed low ability to migrate (Figure 5). LPS141 and FU-DDLS-1 could efficiently invade through matrigel, T778 and SW872 were moderately invasive and SA-4, LISA-2, T449, GOT-3, and T1000 displayed poor ability to invade (Figure 5).

## 4. Discussion

**Figure 6:**
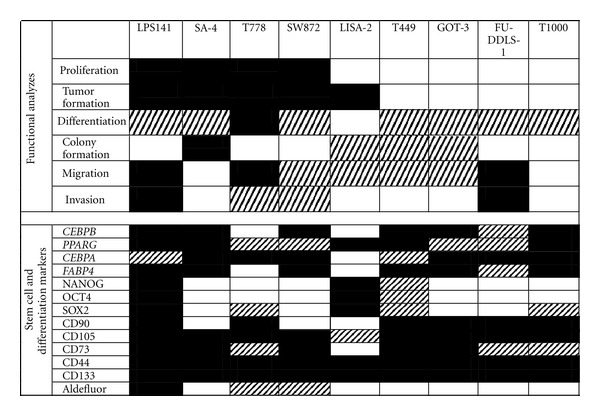
Heat map summarizing the LPS functional analyses and marker expression. Black indicates aggressive signature, grey indicates moderately aggressive signature, and white indicates a nonaggressive signature. Cell lines considered highly aggressive displayed a high proliferation rate; ability to form tumor; resistance towards differentiation; high capacity for colony formation, migration and invasion; low expression of differentiation markers (*CEBPB*, *CEBPA*, *PPARG*, *FABP4*); high expression of pluripotency markers NANOG, OCT4, SOX2; high expression of stem cell surface markers CD90, CD105, CD73, and CD44; expression of cancer stem cell marker CD133 and Aldefluor in more than 3% of the cells. Cell lines considered moderately aggressive displayed the ability to undergo spontaneous differentiation; moderate capacity to form colonies and to migrate or invade; moderate expression of differentiation markers (*CEBPB*, *CEBPA*, *PPARG*, *FABP4*) pluripotency markers NANOG, OCT4, SOX2, and stem cell surface markers CD90, CD105, CD73, and CD44; CD133 and Aldefluor expression in (0.1–3%) of the cells. Cell lines considered non-aggressive displayed a low proliferation rate, did not form tumors *in  vivo*; adipocytic differentiated at basal level; low capacity for colony formation, migration and invasion; high expression of differentiation markers *CEBPB*, *CEBPA*, *PPARG*, *FABP4*; low expression of pluripotency markers NANOG, OCT4, SOX2; expression of stem cell surface markers CD90, CD105, CD73, and CD44 in a low percentage of the cells and undetectable expression of the cancer stem cell markers.

LPS cell lines are valuable model systems for studying liposarcomagenesis and for preclinical investigations. Several studies indicate that the MDM2 antagonist, Nutlin-3a, has therapeutic efficacy against LPS [20–22], but the studies are limited by the small number of cell lines used. The NCI60 cell line panel, used for multiple anticancer drug screens (reviewed [23]) represents the more common cancer types, but does not include a single sarcoma line [24]. Mills et al. (2009) recently generated a cell line panel consisting of 22 sarcoma cell lines, representing 8 histological subtypes [12], but only 4 LPS lines were included. The EuroBoNet consortium has characterized a large number of sarcoma cell lines and xenograft models [13, 25–27], but their focus is on osteosarcoma. The only characterization study dedicated to LPS to date has been performed by Peng et al. (2010), who has undertaken a thorough characterization including analyses of proliferative capacity, migratory and invasive capability, and tumor-forming ability. However, the study focused on primary cell lines, out of which only 4 DDLPS immortal lines were generated [11]. Here, we (The International Liposarcoma Consortium, http://www.liposarcom/aresearch.org/) have characterized 9 immortal LPS cell lines in detail (summarized in Figure 6), and we will continue to extend this panel and utilize it for functional studies.

Tumor formation *in  vivo* is a key property for a cancer cell line. Five of the LPS cell lines formed tumors efficiently in mice, thus being useful for *in  vivo* studies. In comparison to the study by Peng et al., they found that cell lines derived from DDLPS generated tumors in mice, while cell lines derived from WDLPS or from the well-differentiated components of a DDLPS specimen did not generate tumors in SCID mice. We did not find a similar correlation in our study, as several of the WDLPS cell lines generated tumors *in  vivo*. Furthermore, the DDLPS-derived cell line, FU-DDLS-1, appeared nontumorigenic in our NOD-SCID mice. However, FU-DDLS-1 was reported to form tumors in SCID mice when more cells were injected [7]. Tumor-forming ability has been demonstrated to be strain dependent, but the NOD-SCID strain used here is highly immune compromised and considered the most efficient model system for xenotransplantation [28]. Notably, all the cell lines with high proliferative capacity *in  vitro* formed tumors *in  vivo*. We cannot exclude that additional lines can give rise to tumors upon extended periods. However, assays which run for more than 6 months are practically inconvenient.

The stemness and differentiation potential of the LPS cell lines were determined by a number of complementary analyses. LISA-2 was the only cell line staining positive for oil red O and expressing high levels of *C/*EBP*β*, PPAR*γ*, *C/*EBP*α*, and *FABP4* during normal logarithmic growth spontaneously, mimicking the expression pattern of differentiated hMSCs. Surprisingly, T778 expressed the late adipocytic differentiation marker *FABP4 *strongly, indicating active adipogenesis. However, in the functional assay, T778 cells displayed resistance towards induced adipocytic differentiation indicating that the mature differentiated phenotype must be prevented by unknown factors. Thus, it is possible that mutations conferring dedifferentiation may have occurred in a more mature adipocytic cell. In comparison to the study performed by Peng et al., the majority of the cell lines in our panel (8/9) were able to undergo induced adipocytic differentiation, while only a subset of the cell lines analyzed in the latter study underwent adipocytic differentiation. LPS141, which is the only cell line included in both studies, did not undergo induced differentiation in the hands of Peng et al. It is possible that these observed differences are due to the use of different adipocytic differentiation media in the two studies.

Re-expression of the embryonic stem cell and pluripotency factors OCT4, SOX2, and NANOG has been associated with poorly differentiated and aggressive cancers, such as high-grade breast cancer, glioblastomas, and bladder carcinomas [29]. Only LPS141 and LISA-2 expressed all three pluripotency genes highly. Surprisingly, there was no correlation between high OCT4, SOX2, and NANOG expression and colony-forming ability, since LPS141 expressed high mRNA levels of all three pluripotency genes, but did not form colonies readily, while SA-4 displayed high colony-forming capacity, but expressed low levels of the pluripotency transcripts.

Cell surface antigen expression is frequently used as CSC markers. LPS CSCs may arise from hMSCs and we determined the expression of CD90, CD105, CD73, and CD44, all expressed on MSCs [30–32]. We also determined CD133 expression and Aldefluor activity, previously used to enrich CSCs from a LPS model [33]. CD90 expression has not been linked to sarcoma CSCs, but has been associated with tumor-forming ability in liver cancer [34]. The large variation in CD90 expression observed in our panel has also been observed in primary liposarcomas [35]. CD105 was generally expressed in almost all the cells, except in LISA-2 (~20%). The relevance of difference in expression is unclear. CD105 (reviewed [36]) appears to play opposing roles in different cancers, functioning as tumor suppressor in invasive breast cancer, where high expression correlates with improved clinical outcome [37], while high expression correlates with decreased survival in Ewing sarcoma [38]. CD105 function is likely context dependent, and CD105 expression has been associated with procancer function in sarcoma [38, 39]. High CD73 expression has been associated with invasion, metastasis and decreased survival in a range of solid tumors [40–43]. CD73 expression varied greatly in our panel, but no correlation between CD73 expression and invasive capacity was observed. CD44 expression has been demonstrated in a number of sarcoma cell lines and patient material [35, 44]. Although CD44 has not been used as sarcoma CSC marker, it is a well-established CSC marker in other cancers [45–50]. However, CD44 is ubiquitously expressed in the LPS cell lines and thus appears to be irrelevant to the CSC phenotype. CD133 is regarded a CSC-marker in a range of cancers [51–56], including sarcomas of the bone [35, 57], rhabdomyosarcoma [57], and LPS [33]. Interestingly, CD133 was expressed in a subpopulation of all the cell lines, consistent with such a function. The Aldefluor assay is used to enrich CSCs from a range of cancers [5, 33, 50, 58–61], and CSC enrichment is improved when Aldefluor is combined with other markers [33, 50, 59, 62, 63]. We and others have shown that culturing with 10% FBS generally leads to significant reduction in the Aldefluor^high^ population. However, the activity can be rescued or increased by xenotransplantation or serum-free culturing [62]. Since LPS-CSCs are enriched within the Aldefluor^high^CD133^high^ [33], it would be interesting to investigate whether a potential Aldefluor^high^CD133^high^ subpopulation in LPS141, the only line harboring more than 0.2%  Aldefluor^high^ cells, displays CSC characteristics.

## 5. Conclusion

In conclusion, the characterization of the LPS cell lines presented here will support further research on this serious orphan disease and benefit researchers when choosing cell lines for their experimental and preclinical studies. More specifically, the LPS141 is a candidate cell line for testing novel drugs targeting CSCs, displaying tumorigenic and invasive traits, expressing high levels of the pluripotency factors OCT4, SOX2 and NANOG as well as harboring CD133^high^ and Aldefluor^high^ cells. T449 is slow growing, nontumorigenic, noninvasive and able to undergo differentiation, while T778 (from the same patient) is fast growing, tumorigenic, invasive and displays resistance to differentiation and the two lines may serve as models for studying tumor progression and recurrence. T1000 and GOT-3 are the least aggressive cell lines in the panel and suitable models for studying suspected LPS oncogenes by overexpression. We observed a correlation between high proliferation rate and tumor-forming ability *in  vivo*, but there was surprisingly little correlation between the outcome of the different stem cell and differentiation assays, and between the functional assays and the expression of markers.

## Figures and Tables

**Table 1:** *In vivo* tumorigenicity.

Cell line	Tumor formation
LPS141	Yes (6/6)
SA-4	Yes (6/6)
T778	Yes (6/6)
SW872	Yes (11/12)
LISA-2	Yes (6/6)
T449^∗^	Yes? (4/6)^∗+^
GOT-3^∗^	Yes? (10/12)^∗^
FU-DDLS-1	No (0/12)
T1000	No (0/6)

^∗^Small growths, not increasing in size.

Experiments were performed over 6-month period.

^+^Experiment was extended additional 6 weeks.
